# Evaluation of culture media for the production of secondary metabolites in a natural products screening program

**DOI:** 10.1186/2191-0855-3-71

**Published:** 2013-12-17

**Authors:** Karen M VanderMolen, Huzefa A Raja, Tamam El-Elimat, Nicholas H Oberlies

**Affiliations:** 1 Department of Chemistry and Biochemistry, University of North Carolina at Greensboro, Greensboro, NC, USA

**Keywords:** Media, Screening, Secondary metabolites, Fungi

## Abstract

Variation in the growing environment can have significant impacts on the quantity and diversity of fungal secondary metabolites. In the industrial setting, optimization of growing conditions can lead to significantly increased production of a compound of interest. Such optimization becomes challenging in a drug-discovery screening situation, as the ideal conditions for one organism may induce poor metabolic diversity for a different organism. Here, the impact of different media types, including six liquid media and five solid media, on the secondary metabolite production of three fungal strains was examined in the context of the drug-discovery screening process. The relative production of marker compounds was used to evaluate the usefulness and reliability of each medium for the purpose of producing secondary metabolites.

## Introduction

Fungi provide a plentiful and diverse source of unique and often bioactive metabolites, and they have produced a number of medicinally important compounds, including penicillin, mevinolin (Lovastatin) (Gloer, 2007), fingolimod (Strader *et al.*, 2011), and caspofungin (Keating & Figgitt, 2003). The search for new and active compounds from microbial sources is a pursuit for many natural products laboratories. Typically, these efforts will employ a standard culture procedure that most or all microbial strains pass through as a preliminary step to the natural products discovery process. The manipulation of environment and nutrition has been shown to have substantial impacts on the quantity and diversity of secondary metabolite production (Fiedurek *et al.*, 1996; Bode *et al.*, 2002; Miao *et al.*, 2006; Bills *et al.*, 2008; Xu *et al.*, 2008; Mohanty & Prakash, 2009; Kossuga *et al.*, 2012; Shang *et al.*, 2012), and optimization of this initial step, the microbial culture, may have profound effects on the output and success of a natural products screening program.

Methodical modification of culture growing conditions is often referred to as the OSMAC (one strain, many compounds) approach (Bode *et al.*, 2002; Scherlach & Hertweck, 2006). This concept is ideal for fully exploiting the metabolomic diversity of a single or handful of organisms, but it becomes cumbersome when applied to a large-scale screening program. Applying a suite of culture conditions for even one factor, whether it is media, salinity, pH, temperature, etc., to every screened organism quickly becomes so labor and material intensive as to be impractical. The aim of this study, therefore, was to determine an ideal culture medium that could be applied to a drug discovery screening program as a standard preliminary medium.

This ideal medium would have to fit several criteria. In a medium-throughput but academic setting, large volumes of media will be used, so only inexpensive, readily available, and easily prepared media were evaluated. In addition, some media that have shown promise as metabolite inducers in other studies, such as vermiculite (Bills *et al.*, 2012), were excluded due to the high start-up cost necessary for such cultures. Our aim was to evaluate different media that could be integrated into the screening process with minimal changes to the existing equipment available. Six liquid broths and five solid-state media were chosen for evaluation. Three fungal strains were grown on each medium, and the resulting metabolite profiles were assessed.

In addition to a qualitative examination of the metabolite profile, the production of several marker compounds was tracked. Each fungus chosen for this study was previously studied and characterized by our laboratory, and each produced a known compound sharing many structural features with compounds that our research group isolates. Two [*Glomerella acutata* (G2) and a *Hypocreales* sp. (G24)] were isolated as endophytes from pawpaw (*Asimina triloba* L., *Annonaceae*) leaves collected in North America. The third strain, a *Fusicolla* sp. (G142), was isolated from freshwater submerged wood collected in North America. All strains used belonged to *Hypocreales*, of the phylum *Ascomycota* (Kirk *et al.*, 2011). G2 and G142 both produced aurofusarin, while G24 produced PC3. The goal was to identify two distinct fungi, one an endophyte and one from freshwater, that produced identical secondary metabolites, and two from the same source, endophytes from pawpaw, that biosynthesized distinct metabolites. The production of these metabolites was tracked to provide a more in-depth assessment of the fungal extracts.

## Materials and methods

### Isolation of fungi

*Study site, collection and isolation of fungal endophyte strains.* Healthy living twigs and leaves of pawpaw (*Asimina triloba* L., *Annonaceae*) were collected at random from a mature tree located on a private property in Indianapolis, Indiana, USA in July 2011 (9012 Colgate St.). Extra moisture was removed from the samples, and they were transported back to the laboratory in paper bags, where they were processed within 24–48 hours (Stone *et al.*, 2004). In the lab, the twigs and leaves were cut into small pieces (approximately 2–5 mm in length) and washed in distilled water (H_2_O). Subsequently, the segments were surface-sterilized by sequential immersion in 95% ethanol (EtOH; 10 s), sodium hypochlorite (10-15% available chlorine; 2 min), and 70% EtOH (2 min) (Stone *et al.*, 2004). After the sterilization procedure, the plant segments were washed in distilled H_2_O and allowed to dry. The surface-sterilized segments were then transferred using sterile conditions onto 2% malt extract agar [MEA; Difco, 20 g MEA, 1 L sterile distilled H_2_O amended with streptomycin sulfate (250 mg/L) and penicillin G (250 mg/L)]. To test the efficiency of the surface-sterilization procedure, and to confirm that emergent fungal colonies were indeed endophytic and not of epiphytic origin, individual surface-sterilized leaf and stem segments were spread on separate 2% MEA plates with antibiotics; the absence of fungal growth on the nutrient media confirmed the effectiveness of the sterilization procedure (Schulz *et al.*, 1993). Plates were sealed with parafilm and incubated at room temperate in 12 h dark/light cycles until the emergence of fungal colonies was observed. Axenic cultures of emergent fungal endophytes are maintained at the University of North Carolina at Greensboro, Department of Chemistry and Biochemistry Fungal Collection and have been deposited as described below.

*Isolation of fungi from submerged wood in fresh water.* Submerged, dead woody debris was collected randomly in a freshwater lake in North Carolina, USA in October 2011 (Lake Brandt, 36°10′1″N, 19°50′ 18″W). Efforts were made to identify and collect substrates that had been submerged for a considerable time. This was estimated by observation of the degree of softening by fungal soft rot and colonization by other aquatic organisms. Samples were further processed in the laboratory to obtain axenic fungal cultures using established procedures (Shearer *et al.*, 2004; Raja *et al.*, 2009). Axenic cultures of freshwater fungi are maintained at the University of North Carolina at Greensboro, Department of Chemistry and Biochemistry Fungal collection and have been deposited as described below.

*Molecular identification of fungal strains by DNA extraction, PCR and sequencing.* Methods employed to identify fungi isolated in this study using the nuclear ribosomal internal transcribed spacer (ITS) region 1, 2, along with the short structural gene (5.8S) have been outlined in the supporting information of earlier studies (El-Elimat *et al.*, 2013b; El-Elimat *et al.*, 2013c; Figueroa *et al.*, 2013). The ITS region was chosen to identify fungal species, as it has been recently identified as a barcode marker for fungi (Schoch *et al.*, 2012). Sequence data were deposited in GenBank; the accession numbers for G2, G24, and G142 were AB858344, AB858345, and AB858346, respectively. Cultures were deposited with the Leibniz Institute DSMZ-German Collection of Microorganisms and Cell Cultures; the accession numbers for G2, G24, and G142 were DSM 27861, DSM 27862, and DSM 27863, respectively.

### Liquid media cultivation

Several liquid media were chosen to represent a broad range of nutrient sources, including both defined and undefined broths. Each fungal strain was first cultivated on 2% malt extract agar. Plugs from these cultures were used to inoculate 50 mL of six different culture broths in 250 mL Erlenmeyer flasks. These broths included Czapek Dox (CD; Sigma Aldrich), 2% Malt Extract (ME; Difco), Potato Dextrose (PD; Difco), YPSS (4 g yeast extract, 14 g soluble starch, 1 g dibasic K_2_HPO_4_, 0.5 g MgSO_4_•7H_2_O, 1 L H_2_O), YESD (20 g soy peptone, 20 g dextrose, 5 g yeast extract, 1 L H_2_O), and PYG (1.25 g soy peptone, 1.25 g yeast extract, 5 g D-glucose, 1 L H_2_O). Liquid cultures were grown for 14 days on an orbital shaker (100 rpm) at room temperature before extraction. Each culture was grown in triplicate.

### Solid media cultivation

Five forms of solid media, including rice, grits, oatmeal, wheat germ, and a 3:1:1:1 mixture of the same were used. For the preparation of solid media, 10 g of medium were mixed with 25 mL of deionized H_2_O in a 250 mL Erlenmeyer flask, sealed with a foam plug and tinfoil, and autoclaved. Plugs (from the 2% malt extract agar cultures listed above) of each fungus were used to inoculate 15 mL of YESD. These seed cultures were grown for three days on an orbital shaker (100 rpm) at room temperature, and these were used to inoculate the solid media, which was allowed to grow (stationary) for an additional 11 days at room temperature. Each culture was grown in triplicate.

### Extraction of fungal cultures

To each culture, 60 mL of 1:1 chloroform:methanol (CHCl_3_:MeOH) were added. Solid media cultures were chopped with a spatula to ensure proper mixing. The cultures were shaken overnight (100 rpm) at room temperature and subsequently filtered by vacuum. The filtrate was stirred for 1 h with 90 mL of CHCl_3_ and 100 mL deionized H_2_O. This mixture was then transferred to a separatory funnel, and the bottom organic layer was evaporated to dryness *in vacuo*. The dried organic extract was reconstituted with 100 mL 1:1 acetonitrile (CH_3_CN):MeOH and 100 mL hexanes. This biphasic solution was stirred for 1 h and transferred to a separatory funnel. The bottom, defatted organic extract was evaporated to dryness *in vacuo.*

### Analysis of fungal metabolite profiles by ultra performance liquid chromatography-mass spectrometry (UPLC-MS)

**Figure 1:**
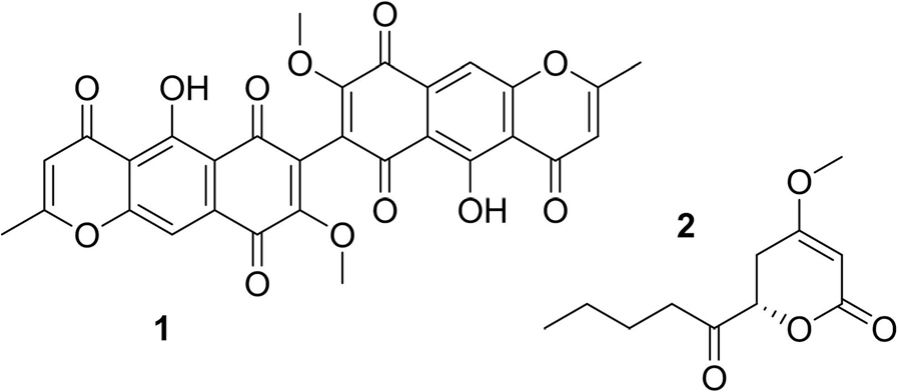
Marker compounds used to track the production potential of various media; (1) aurofusarin, (2) PC3.

The defatted organic extract of each fungal culture was examined by UPLC-MS. The method used was developed as part of a dereplication procedure (El-Elimat *et al.*, 2013a). In brief, analyses were performed using a Waters Acquity UPLC system utilizing a BEH C18 column (Waters; 50 mm × 2.1 mm i.d., 1.8 μm). The mobile phase consisted of CH_3_CN and 0.1% formic acid-H_2_O, increasing linearly from 15% CH_3_CN at the time of injection to 100% CH_3_CN at 10 min. The flow rate was 0.3 mL/min and column temperature was 40°C. The UPLC system was coupled to a Thermo Scientific LTQ Orbitrap XL equipped with electrospray ionization (ESI) source. The production of several known metabolites (Figure 1) was tracked using high-resolution mass spectrometry (HRMS). Crude extracts were dissolved in 1:1 MeOH:dioxane at a concentration of 2.0 mg/mL; 6.0 μL were injected, and the peak areas of each metabolite measured. Additionally, extracts were also examined using the same UPLC system equipped with an evaporative light scattering detector (ELSD; Waters); all other conditions were the same.

## Results

### Effects of media on fungal growths

**Figure 2:**
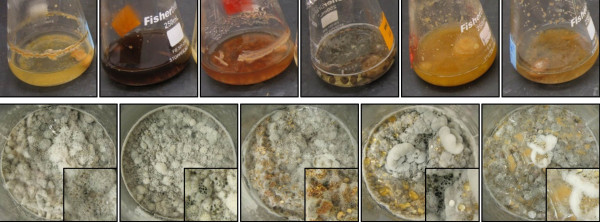
**Photos of G2 in liquid media (top); from left to right: Czapek dox, 2% malt extract, potato dextrose, YPSS, YESD, and PYG broths.** Photos of G2 on solid media (bottom); from left to right: rice, grits, oatmeal, wheat germ, and 3:1:1:1, respectively, of the same. Enlargements of the photos (2.5×) are provided in the lower right hand corner to help visualize the production of spores (orange) and/or exudates (black).

The visual differences in fungal growth were the most immediately apparent result of varying the culture medium and were a primary instigator of this study; striking differences in color occurred as various secondary metabolites were stimulated by a new medium (Figure 2). These color changes were most notable in liquid media, but the amount of sporulation (orange) and exudate (black) produced varied between solid media, too. As an example, after the 11 days of growth on solid media, the rice cultures of G2 exhibited almost no sporulation or exudate (Figure 2), while the grits culture showed a small amount of black exudate, the oatmeal culture exhibited heavy sporulation (orange), and the wheat germ culture had a large amount of both.

### Effects of media on the profile of secondary metabolites

**Figure 3:**
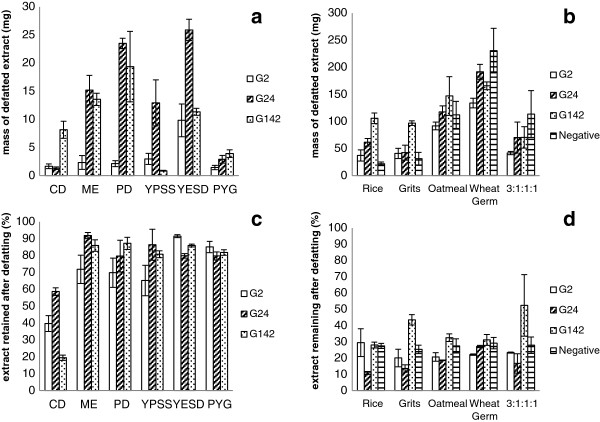
**A comparison of the (a) mass of defatted extract of fungal growths in liquid media, (b) mass of defatted extract of fungal growths on solid media, (c) percentage of organic extract retained after the defatting step (liquid media), and (d) the percentage of organic extract retained after the defatting step (solid media).** Data plotted are means ± SD of three replicate growths per culture medium. The “Negative” series in solid media (b and d) refers to the mass of extracted solid media alone, with no fungal growth. The same data for liquid cultures were negligible and therefore not shown. Abbreviations of media are defined in the Methods section.

The various media tested exhibited marked differences in the mass of extract (Figure 3a and b). On the whole, the cultures grown on solid media produced extracts with masses one to two orders of magnitude larger than the same fungus grown in any of the liquid media. In both solid and liquid cultures, G2 consistently produced the lowest mass of extract, though this difference was more notable in the liquid media. The variation of the amount of extract produced was greater in liquid media than solid. The greatest variation between liquid media was shown by G24; in Czapek Dox broth, the culture produced less than two mg of total defatted extract, while it produced more than 20 mg when grown in potato dextrose broth and YESD broth (Figure 3a).

The mass of the defatted extract was also compared to the mass of the extract prior to the defatting step of the extraction procedure (Figure 3c and d). Defatting is an important step in the extraction process for two reasons. The hexane wash eliminates some common but unwanted metabolites, such as fatty acids, that are not of interest to most natural product screening programs. It also cleans the extract of the very nonpolar constituents that are detrimental to reverse phase columns. Analyzing the percentage of extract lost in this defatting step can help to characterize the composition of the fungal extract.

Extracts from solid media cultures retained a much smaller percentage of the original mass, typically between 10 and 30%, though G142 retained higher percentages of 42 and 52% on grits and the 3:1:1:1 mixture, respectively. Growths on liquid media, on the other hand, retained between 70 and 90% of their mass after the defatting wash, though all three fungi grown on Czapek Dox lost significant portions of their extract in this step.

### Effects of media on composition of extract

The UPLC profiles of the fungal extracts showed wide variation between media (Additional file 1). Particularly in the liquid cultures, metabolites with vastly different retention times were produced. Grown in 2% malt extract broth (ME), G142 produced nine major peaks that eluted between 4 and 6.5 min on a 10-min CH_3_CN gradient. The same fungus grown in YPSS produced only five major peaks that all eluted after 7.5 min (Additional file 1).

The production of marker compounds (Figure 1) was compared between media by measuring the peak areas of each metabolite via UPLC-MS and multiplying by total mass of the defatted extract, in order to more accurately compare the total production of each metabolite instead of the percentage of extract the metabolite comprised. This value was expressed as relative percentage of metabolite production, normalized to the largest value of each metabolite’s set. Aurofusarin was the metabolite of interest in both G2 and G142, while the metabolite of interest produced by G24 was PC3. Aurofusarin is a mycotoxin and red pigment common in *Fusarium* species (Ezekiel *et al.*, 2012; Soerensen *et al.*, 2012; Mikusova *et al.*, 2013). PC3 is an analog of pestalotin, which was first described as a product of an unidentified fungus in 1973 (Strunz *et al.*, 1974); pestalotins synergistically augment the plant hormone gibberellin (Kimura *et al.*, 1971; Kimura *et al.*, 1977; Kirihata *et al.*, 1996). While the structural diversity of compounds from fungi can be vast (El-Elimat *et al.*, 2012), these were chosen as representative compounds for a drug screening program, since they contain heteroatoms, conjugated rings, carbonyl groups, hydroxy, methoxy, and methyl moieties, and polyketide derived chains.

**Figure 4:**
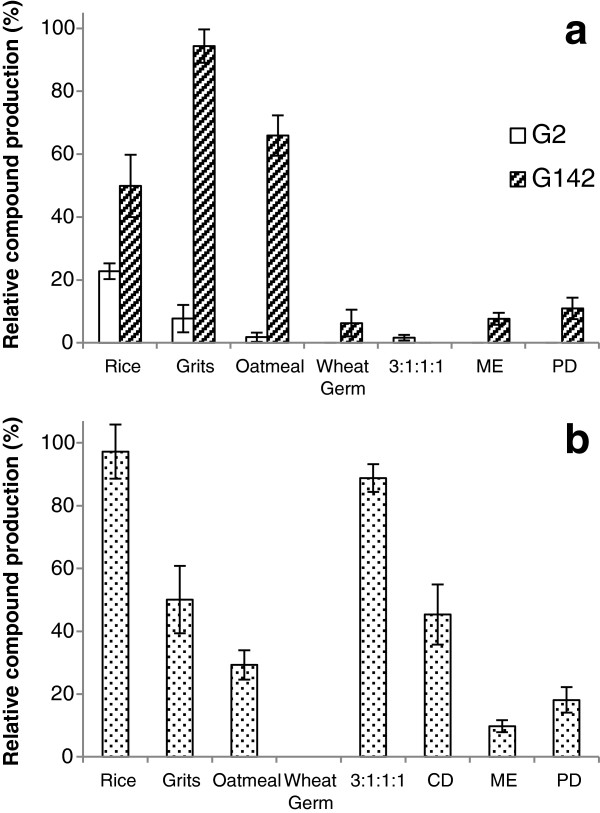
**Relative production of marker compounds aurofusarin in G2 and G142 (a), and PC3 in G24 (b).** Data plotted are means ± SD of three replicate growths per culture medium.

For all fungi, rice and grits consistently produced greater quantities of the marker compounds, while wheat germ consistently produced little or no marker compound. Few of the liquid cultures produced meaningful quantities of the marker compounds (Figure 4), though they were detectable in most (Table 1). G2 produced modest amounts of aurofusarin when grown on oatmeal, when compared to the production when grown on rice; however, G142 consistently produced much greater quantities of aurofusarin on rice, grits, and oatmeal. While G2 produced little or no aurofusarin in the liquid cultures, G142 produced quantities in ME and PD comparable to the production by G2 on several solid cultures (Figure 4). PC3 production was highest when G24 was grown on rice and the 3:1:1:1 mixture, though its production in CD, ME, and PD were similar to the production on solid media.

**Table 1:** Summary of the production of marker compounds in solid and liquid media

	**Rice**	**Grits**	**Oatmeal**	**Wheat germ**	**3:1:1:1**	**CD**	**ME**	**PD**	**YPSS**	**YESD**	**PYG**
Aurofusarin (G2)	m	m	m	d	m	u	d	d	d	u	u
PC3 (G24)	m	m	m	d	m	m	m	m	d	u	u
Aurofusarin (G142)	m	m	m	m	d	d	m	m	d	d	m

Table 1 Detection of marker compounds in solid and liquid media extracts; m = measured, as shown in bar graphs (Figure 4), d = detected at low levels, u = undetected. Limit of detection 0.22 mg/g extract.

## Discussion

It has been shown that media conditions have varying effects on the production of fungal secondary metabolites (Bode *et al.*, 2002; Miao *et al.*, 2006). Often, the variation of culture conditions is used to optimize the yields of a specific compound, such as the active metabolite in a medicinal fungus (Xu *et al.*, 2008) or drug-producing microbe (Pu *et al.*, 2013). In other cases, the OSMAC approach can be used to fully exploit the biodiversity of a small number of microbes, usually fewer than five (Hestbjerg *et al.*, 2002; Paranagama *et al.*, 2007; Shang *et al.*, 2012). These techniques can be very valuable for manipulating the metabolomes of several fungi at a time, but they also increase the manpower necessary to process a single culture by several fold for each growth condition being manipulated. Our laboratory processes approximately 400 new fungal cultures every year; applying the OSMAC approach to each and every culture would be impractical without the implementation of specialized equipment and procedures for the parallel cultivation of screened fungi (Bills *et al.*, 2008).

This study was designed to evaluate media with the intention of optimizing a drug-discovery screening program, which is a different goal than finding the ideal medium for a specific microbial strain producing one or two key metabolites. What may be the best medium for one fungus could often prove unproductive for other strains. For example, G2 produced very little aurofusarin when grown on oatmeal, while G142 produced quantities equivalent to its production on rice. In potato dextrose broth, G24 and G142 produced large extracts, but the extract of G2 was less than 2 mg. Similarly, media that facilitate increased fungal growth and large amounts of extract may not produce compounds of interest. G24, when grown in PD or YESD liquid broths, produced extracts of similar masses to both each other and to its extracts when grown on rice, yet the metabolite of interest (PC3), was produced in significantly smaller quantities in PD and undetected in YESD. Thus, media in this study were evaluated with respect to both the amount of extract produced and the composition of that extract, including the production of the chosen marker compounds (Figure 1). Because the media were being assessed for use in a screening program, consistency of production across all three fungal strains was a more important criterion than excellent production in just one of the strains.

The variation in extract composition and marker compound production was more pronounced than expected when this study was designed. The most surprising outcome was that the marker compounds were undetected by UPLC-MS in several of the liquid culture extracts (Table 1). This was surprising, given that the limit of detection for the method was approximately 0.22 mg/g extract (El-Elimat *et al.*, 2013a). Indeed, the amount of extract produced was often so drastically smaller than those produced by solid media cultures, that for a drug-discovery screening program, it would be impractical to use liquid cultures as a standard culture procedure; isolating sufficient quantities of compounds of interest would be challenging. While it was enticing that the liquid cultures produced a lower percentage of fats than the solid media cultures, since fats complicate the purification process (Figure 3c and d), the overall paucity of metabolites negated this benefit.

The production of marker compounds varied widely between all cultures. On oatmeal, G142 (isolated from freshwater) produced amounts of aurofusarin similar to cultures grown on rice; G2 (an endophyte) had very poor production on oatmeal. The use of only G142 in such a media survey would indicate that oatmeal may be an ideal candidate as a standard solid medium, while the added information provided by using the second fungus producing the same compound refuted this conclusion. A further example was the production of PC3 by G24 when grown on the 3:1:1:1 mixture. Again, while PC3 was produced in quantities similar to that of the rice or grits media, G2 and G142 showed poor aurofusarin production on this same medium. In general, rice appeared to more consistently produce high quantities of the metabolites of interest, while wheat germ consistently did not.

For the purposes of a fungal metabolite screening program, it seems clear that rice would serve well as a standard culture medium. The data supporting the use of rice as an ideal medium may not be surprising to researchers who routinely culture fungi; this study validates the empirical knowledge of many mycologists and natural products researchers who use rice as a go-to culture substrate. While liquid media did not perform well in this study, they should not be discounted as a viable culture condition in other circumstances. Different microbes, such as those found in marine environments, may be far more suited to a liquid growing environment than those cultured here. However, new metabolites have been reported when marine fungi have been grown on rice (Shang *et al.*, 2012), which supports the use of complex solid media as a standard culture condition.

## Competing interests

The authors declare that they have no competing interests.

## Supplementary Material

Additional file 1 **UPLC-ELSD chromatograms of G142 in liquid media.** The column was a BEH-C18, and the gradient increased linearly from 15:85 CH3CN:H2O to 100:0 over 10 min. Abbreviations of media are defined in the Methods section. Click here for file
