# Supplementary material for: Evaluation of culture media for the production of secondary metabolites in a natural products screening program
Source: AMB Express. 2013 Dec 17;3:71. doi: 10.1186/2191-0855-3-71 (PMC3917616; doi:10.1186/2191-0855-3-71)
Supplement: Additional file 1 — UPLC-ELSD chromatograms of G142 in liquid media. The column was a BEH-C18, and the gradient increased linearly from 15:85 CH3CN:H2O to 100:0 over 10 min. Abbreviations of media are defined in the Methods section. [file 2191-0855-3-71-S1.pdf]

Supplementary Material

AMB Express

Evaluation of Culture Media for the Production of Secondary Metabolites in a Natural Products Screening Program

Karen M. VanderMolen, Huzefa A. Raja, Tamam El-Elimat, Nicholas H. Oberlies*

Department of Chemistry and Biochemistry, University of North Carolina at Greensboro, Greensboro, NC, USA

***** nicholas_oberlies@uncg.edu, 336-334-5474

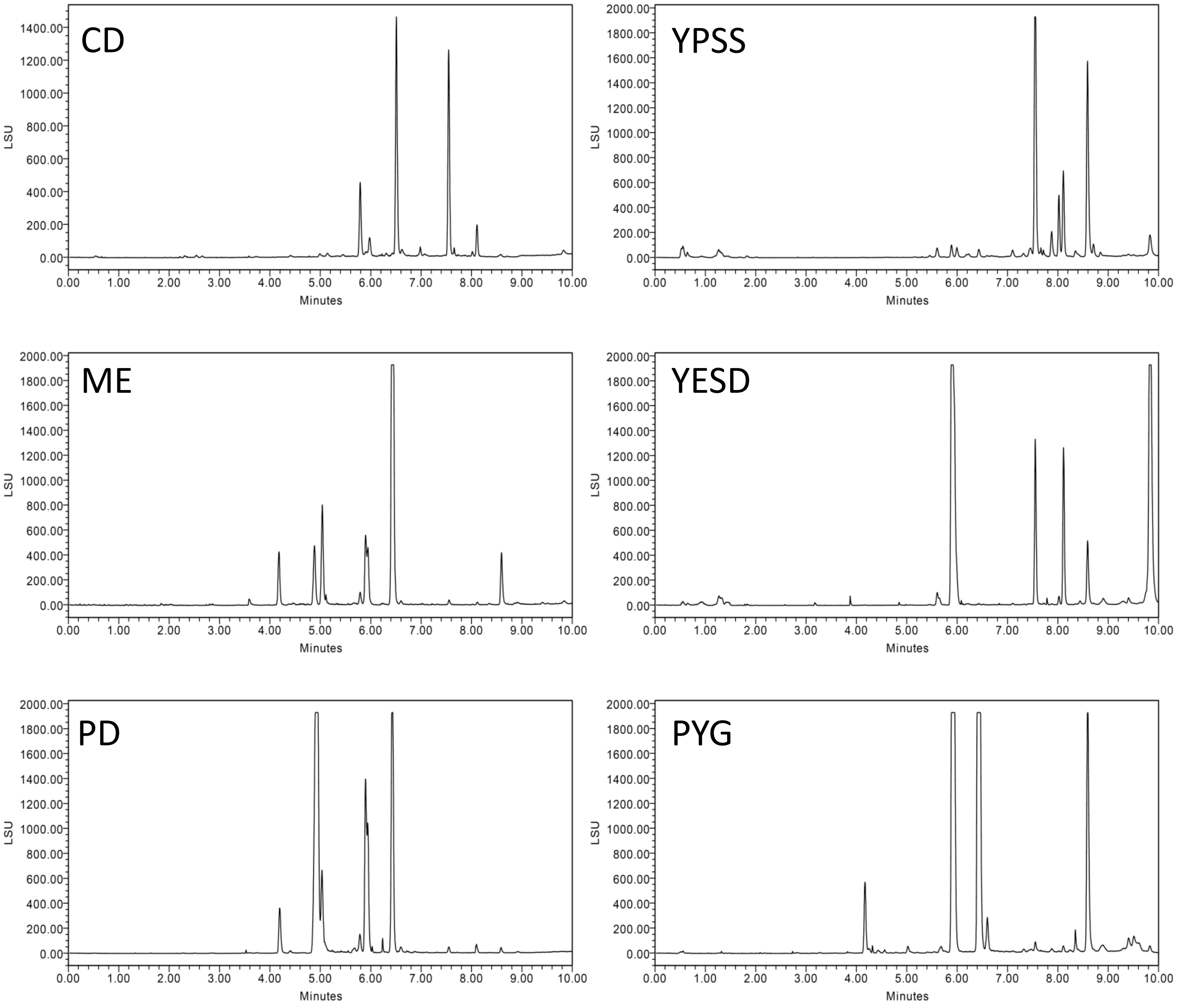

Online Resource 1. UPLC-ELSD chromatograms of G142 in liquid media. The column was a BEH-C18, and the gradient increased linearly from 15:85 CH_3CN:H_2O to 100:0 over 10 min. Abbreviations of media are defined in the Methods section.
